# Correction: Grabež et al. Seaweed Inclusion in Finishing Lamb Diet Promotes Changes in Micronutrient Content and Flavour-Related Compounds of Raw Meat and Dry-Cured Leg (Fenalår). *Foods* 2022, *11*, 1043

**DOI:** 10.3390/foods11172624

**Published:** 2022-08-30

**Authors:** Vladana Grabež, Elena Coll-Brasas, Elena Fulladosa, Elin Hallenstvedt, Torunn Thauland Håseth, Margareth Øverland, Per Berg, Alemayehu Kidane, Bjørg Egelandsdal

**Affiliations:** 1 Faculty of Chemistry, Biotechnology and Food Science, Norwegian University of Life Sciences, NO-1430 Ås, Norway; 2 IRTA, Food Technology and Food Safety Programs, Finca Camps i Armet, E-17121 Monells, Spain; 3 Nortura SA, Økern, NO-0513 Oslo, Norway; 4 Animalia, Økern, NO-0513 Oslo, Norway; 5 Faculty of Bioscience, Norwegian University of Life Sciences, NO-1432 Ås, Norway

## Addition of an Author

“**Alemayehu Kidane**” was not included as an author in the original publication [1]. The corrected Author Contributions Statement appears here. The authors apologize for any inconvenience caused and state that the scientific conclusions are unaffected. This correction was approved by the Academic Editor. The original publication has also been updated.
